# Supplementary material for: Superconducting and normal-state anisotropy of the doped topological insulator Sr0.1Bi2Se3
Source: Sci Rep. 2018 May 16;8:7666. doi: 10.1038/s41598-018-26032-0 (PMC5956080; doi:10.1038/s41598-018-26032-0)
Supplement: Supplementary file 1 — Supplemental Information [file 41598_2018_26032_MOESM1_ESM.pdf]

Supplemental Information

Superconducting and normal-state anisotropy of the doped topological insulator $Sr_{0.1}Bi_2Se_3$

M. P. Smylie,[1,2] K. Willa,[1] H. Claus,[1] A. E. Koshelev,[1] K. W. Song,[1] W.-K. Kwok,[1] Z. Islam[3], G. D. Gu[4], J. A. Schneeloch,[4,5] R. D. Zhong,[4,6] and U. Welp[1]

[1]Materials Science Division, Argonne National Laboratory, Argonne, IL 60439, USA
[2]Department of Physics, University of Notre Dame, Notre Dame, IN 46556, USA
[3]Advanced Photon Source, Argonne National Laboratory, Argonne, IL 60439, USA
[4]Condensed Matter and Materials Science Department, Brookhaven National Laboratory, Upton, NY 11793, USA
[5]Department of Physics and Astronomy, Stony Brook University, Stony Brook, NY 11794, USA
[6]Department of Materials Science and Engineering, Stony Brook University, Stony Brook, NY 11794, USA

In order to rule out doping variations as a cause of the difference in anisotropy seen using magnetotransport and magnetization measurements, we present determinations of the superconducting phase diagram of $Sr_{0.1}Bi_2Se_3$ crystal #5 using both techniques on the same sample.

Magnetoresistance:
$Sr_{0.1}Bi_2Se_3$ crystal #5 is bar-shaped, oriented such that the current flows along the a-direction. Using the 50% criterion, a zero-field T_c of 3.04K is obtained for this sample. The resistive transitions in fields applied along the *a*, *a** and *c*-directions are shown in Fig. S1. With increasing field, the transitions shift uniformly to lower temperature similar to those obtained on crystal #2 displayed in Fig. 3 of the article. The emergence of a substantial in-plane anisotropy is evident from a comparison of panels (a) and (b).

Magnetization:
The temperature dependence of the magnetization measured in several fields along the *a*, *a** and *c* directions and a direct comparison of the 4 kG data for all three orientations are shown in Fig. S2. These data were taken on increasing temperature after cooling the sample in the indicated fields. Also included in Fig. S2(a) are the data in 2 kG obtained on warming after the sample has been cooled in zero-field, showing that the magnetization is essentially reversible.
Near the transition, the magnetization is well described by a linear temperature dependence as indicated by the black lines. This dependence for the equilibrium

magnetization is expected on the basis of Ginzburg-Landau theory and allows for a determination of $T_c(H)$ from the intersect with the m=0 line. The data in Fig. S2(d) clearly reveal the in-plane anisotropy in $T_c(H)$ and in the slopes of $m(T)$, in agreement with Fig. 5a in the article.

Phase diagram:
The phase boundaries deduced from the resistive 50% criterion are shown in Fig. S1(d) as solid circles. The general features of this phase diagram, namely the large in-plane anisotropy and a pronounced upward curvature near T_c, are similar to those in Fig. 3(d) of the article. The anisotropy deduced from these data decreases slightly with increasing temperature from $\Gamma \sim 5.3$ at 2.68 K to 5.15 at 2.8 K and 4.9 at 2.9 K. The shaded areas in Fig. S2(d) are bounded by the H_{c2}-lines obtained with the 2% and 98% criterion corresponding to values close to the zero-resistance point and the resistive onset, respectively. We note that the phase boundaries shift considerably depending on the criterion employed. However, the effect of the criterion is asymmetric for the a and a^* directions reflecting the observation that upon increasing field a tail starts developing for H // a, whereas for H // a^* there appears rounding near the top of the transition. At the same time, the in-plane anisotropy changes from 3.1 for the 2% - criterion, to 5.15 at 50% and 5.0 at 98 %.
Fig. S2(d) also includes the $T_c(H)$ values obtained from the magnetization data. The error bars reflect the uncertainties in locating $T_c(H)$, see Figs. S2(a, b, c). In contrast to the resistive data, the magnetic data yield essentially linear phase boundaries with slopes of 24.6 kG/K and 9.5 kG/K for the a and a^* directions, respectively, corresponding to an in-plane anisotropy of $\Gamma \sim 2.6$. The same value for the in-plane anisotropy is also obtained from the slopes of the $m(T)$-data in Fig. S2(d). We note that the magnetically determined phase boundaries for the a^* and c directions almost coincide, in agreement with the resistive data. The unusual situation arises that the magnetically determined phase boundary lies above the resistive 50%-line for H // a^* whereas it lies below for H // a implying that there is no criterion that would bring both data sets in alignment. The reasons for this unexpected behavior are not understood at present, and may be related to the unusual positive curvature of the resistively determined H_{c2} observed in all samples, or to the existence of surface states, which may have different superconducting properties than the bulk.

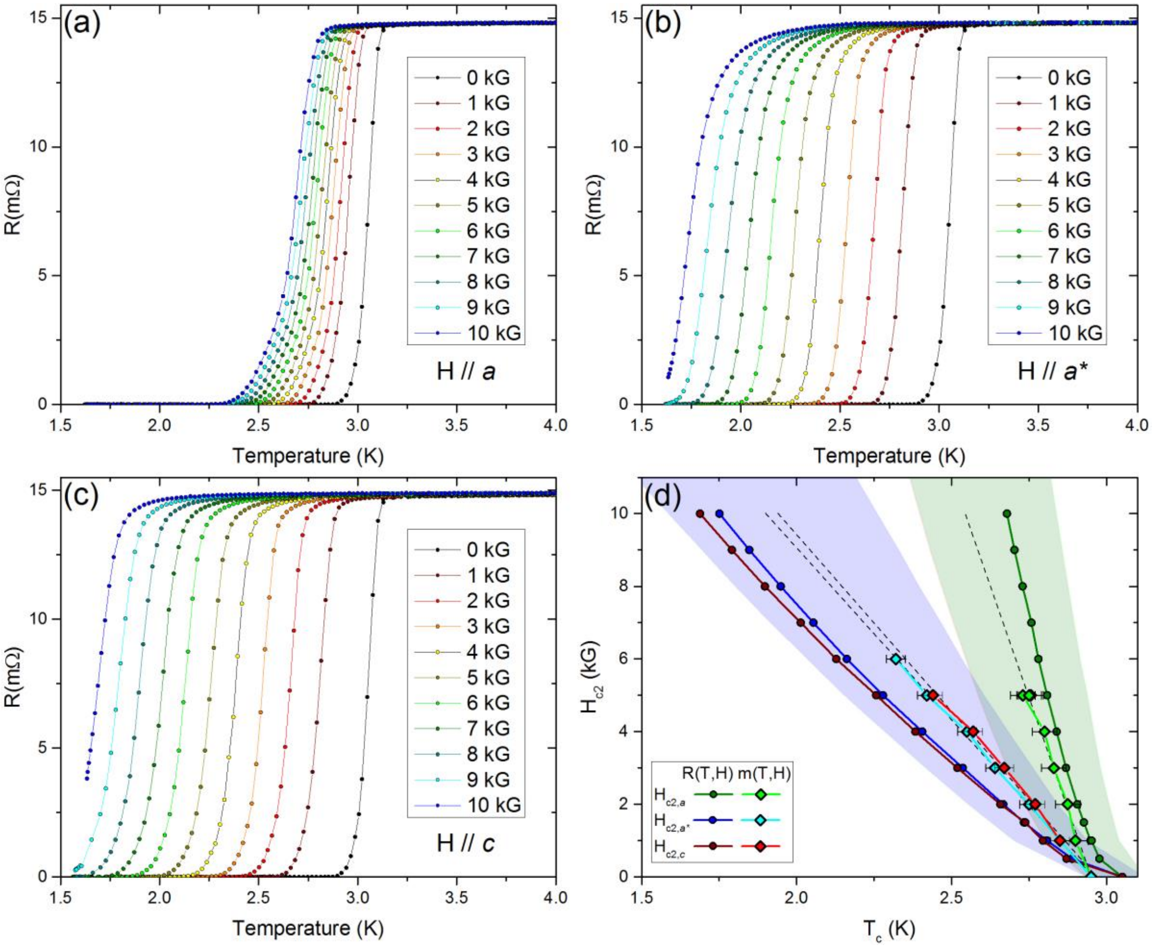

Fig. S1: Temperature dependence of the resistance of $Sr_{0.1}Bi_2Se_3$ crystal #5 measured in various magnetic fields applied along the principal crystal directions. (a) Field vector H // a. (b) Field vector H // a^*. (c) Field vector H // c. (d) Superconducting phase diagram; circles are from R(T,H), diamonds are from m(T,H). The two shaded regions mark the phase boundaries for the resistive 2% and 98% criterion for the a (green) and a^* (blue) directions. For each, the 2% criterion has the lower H_{c2}.

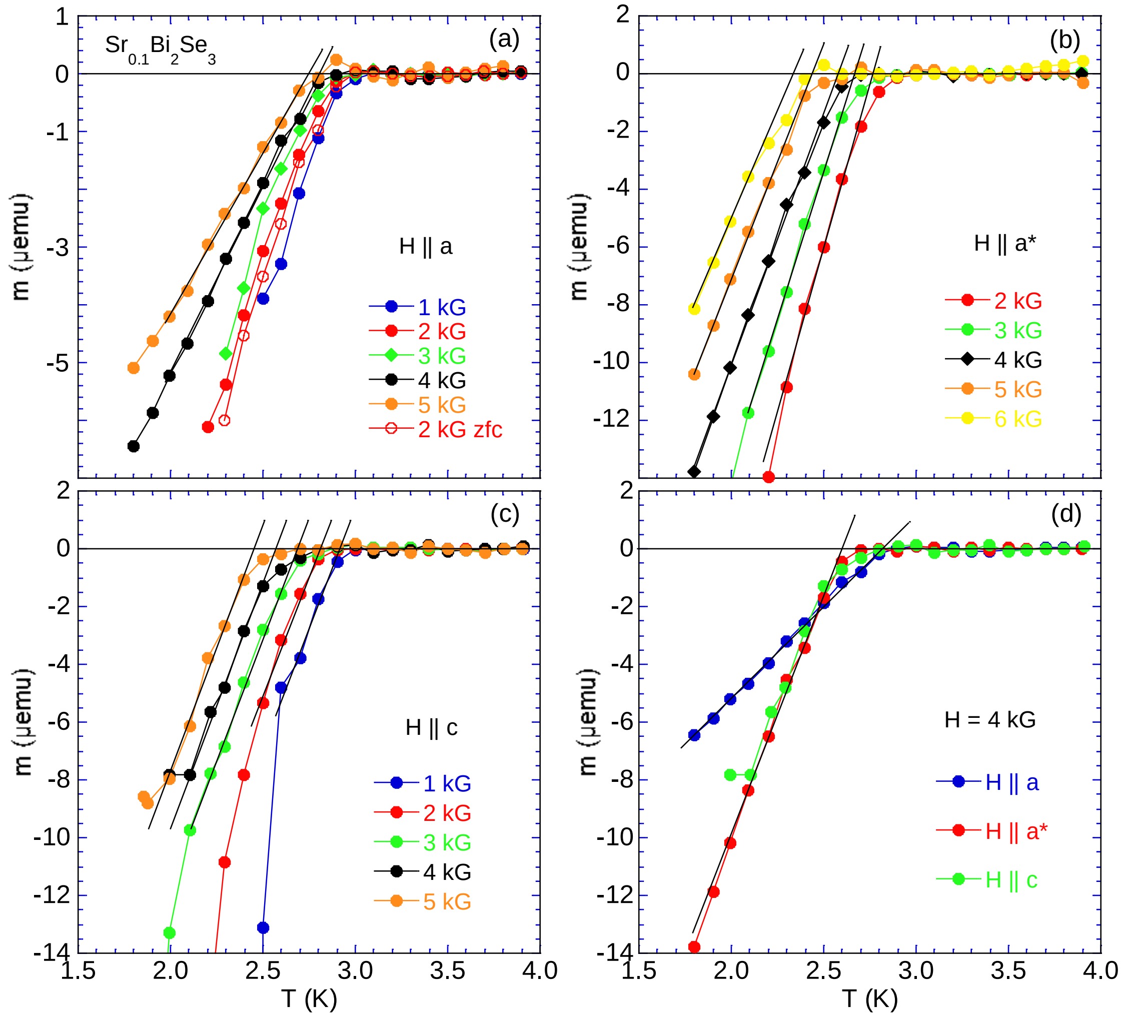

Fig. S2: Temperature dependence of the magnetic moment of $Sr_{0.1}Bi_2Se_3$ crystal #5 measured in various magnetic fields applied along the principal crystal directions. The black lines mark the almost linear temperature dependence of the magnetic moment near T_c. (a) Field vector H // a. (b) Field vector H // a^*. (c) Field vector H // c. (d) Comparison of the 4-kG data for all three orientations.
